# The Unforeseen Non-Coding RNAs in Head and Neck Cancer

**DOI:** 10.3390/genes9030134

**Published:** 2018-03-01

**Authors:** Alexandra Iulia Irimie, Alina-Andreea Zimta, Cristina Ciocan, Nikolay Mehterov, Diana Dudea, Cornelia Braicu, Ioana Berindan-Neagoe

**Affiliations:** 1 Department of Prosthetic Dentistry and Dental Materials, Division Dental Propaedeutic, Aesthetic, “IuliuHatieganu” University of Medicine and Pharmacy, Cluj-Napoca, 23 Marinescu Street, 40015 Cluj-Napoca, Romania; irimie.alexandra@umfcluj.ro (A.I.I.); ddudea@umfcluj.ro (D.D.); 2 MEDFUTURE—Research Center for Advanced Medicine, University of Medicine and Pharmacy Iuliu-Hatieganu, 23 Marinescu Street, 40015 Cluj-Napoca, Romania; andreea.zimta@umfcluj.ro (A-A.Z.); cristina.ciocan@umfcluj.ro (C.C.); 3 Department of Medical Biology, Medical University Plovdiv, BulVasilAprilov 15-А, Plovdiv 4002, Bulgaria; ni_ki82@abv.bg; 4 Technological Center for Emergency Medicine, BulVasilAprilov 15-А, Plovdiv 4002, Bulgaria; 5 Research Center for Functional Genomics and Translational Medicine, “IuliuHatieganu” University of Medicine and Pharmacy, 23 Marinescu Street, 40015 Cluj-Napoca, Romania; braicucornelia@yahoo.com; 6 Department of Functional Genomics and Experimental Pathology, The Oncology Institute “Prof. Dr. Ion Chiricuta”, Republicii 34 Street, 400015 Cluj-Napoca, Romania

**Keywords:** head and neck cancer, rare non-coding RNAs, biological mechanisms

## Abstract

Previously ignored non-coding RNAs (ncRNAs) have become the subject of many studies. However, there is an imbalance in the amount of consideration that ncRNAs are receiving. Some transcripts such as microRNAs (miRNAs) or small interfering RNAs (siRNAs) have gained much attention, but it is necessary to investigate other “pieces of the RNA puzzle”. These can offer a more complete view over normal and pathological cell behavior. The other ncRNA species are less studied, either due to their recent discovery, such as stable intronic sequence RNA (sisRNA), YRNA, miRNA-offset RNAs (moRNA), telomerase RNA component (TERC), natural antisense transcript (NAT), transcribed ultraconserved regions (T-UCR), and pseudogene transcript, or because they are still largely seen as non-coding transcripts with no relevance to pathogenesis. Moreover, some are still considered housekeeping RNAs, for instance small nucleolar RNAs (snoRNAs) and TERC. Our review summarizes the biogenesis, mechanism of action and potential role of less known ncRNAs in head and neck cancer, with a particular focus on the installment and progress for this particular cancer type.

## 1. Introduction

Head and neck cancer encompasses a group of malignancies developing in the areas of the head outside the central nervous system [1]. Esophageal cancer is associated with head and neck cancer due to its anatomical proximity and common risk factors. The major risk factors are increased alcohol consumption, tobacco smoking, human papilloma virus (HPV) infection and betel chewing [2,3,4,5]. 

Squamous cell carcinoma is related to *NOTCH1*, *PIK3CA* and *PTEN* mutations, while esophageal cancer is related to high rates of *KRAS* mutations and *HER2* amplification [6]. Accompanying these mutations, there are alterations in the transcriptomics landscape of the cells [7].

The present study is an incursion on rare RNA. Their biogenesis, mechanism of action and biological effects are analyzed, in relation to their implication in head and neck cancer [8,9,10,11,12]. The importance of microRNAs (miRNAs) and small interfering RNAs (siRNAs) cannot be ignored [9,13]. It is however important to study other molecular players of carcinogenesis that would offer a more complete view [13,14]. Each class of these lesser known transcripts is presented separately, with a particular focus on the relationship with head and neck cancer. 

## 2. The Unforeseen RNAs

A few decades ago, all molecular biology studies were based on the central dogma that stated there is an exchange of information between DNA and RNA, and that this unequivocal information encodes the protein sequence of amino acids [10,15,16]. With the discovery of miRNAs in *Caenohabdilis elegans* and small interfering RNAs (siRNAs) in plants [17,18,19], the central dogma was challenged. This was the first time that the issue of non-coding RNAs (ncRNAs) with regulatory functions entered the mainstream of molecular research [17,18,19]. RNAs are nowadays classified as protein-coding RNAs and ncRNAs. Nevertheless, some RNAs were found in the grey area, as having both regulatory and protein-coding abilities [20]. 

miRNAs are the most studied transcripts. Thus far, there are numerous preclinical studies that are trying to formulate better diagnostic, prognostic and therapeutic options based on miRNAs [10,14,21,22,23,24,25,26]. The miRNAs that are up-regulated in cancer cells are called oncomiRs. The down-regulated miRNAs are named tumor suppressor miRNAs [27]. In addition, there is an abundance of data regarding the role of miRNAs in the diagnosis and prognosis of head and neck cancer [7,28].

siRNAs are also a well-known ncRNAs class. These transcripts are used in preclinical study as therapeutic strategy or to study the role of a specific gene, mainly due to their ability to target a single gene [13,29,30,31]. siRNAs can also become the basis for future therapeutic strategies, targeting cancer-promoting gene transcripts in various cancers [29,31,32,33,34,35], such as head and neck cancer [36,37].

**Figure 1:**
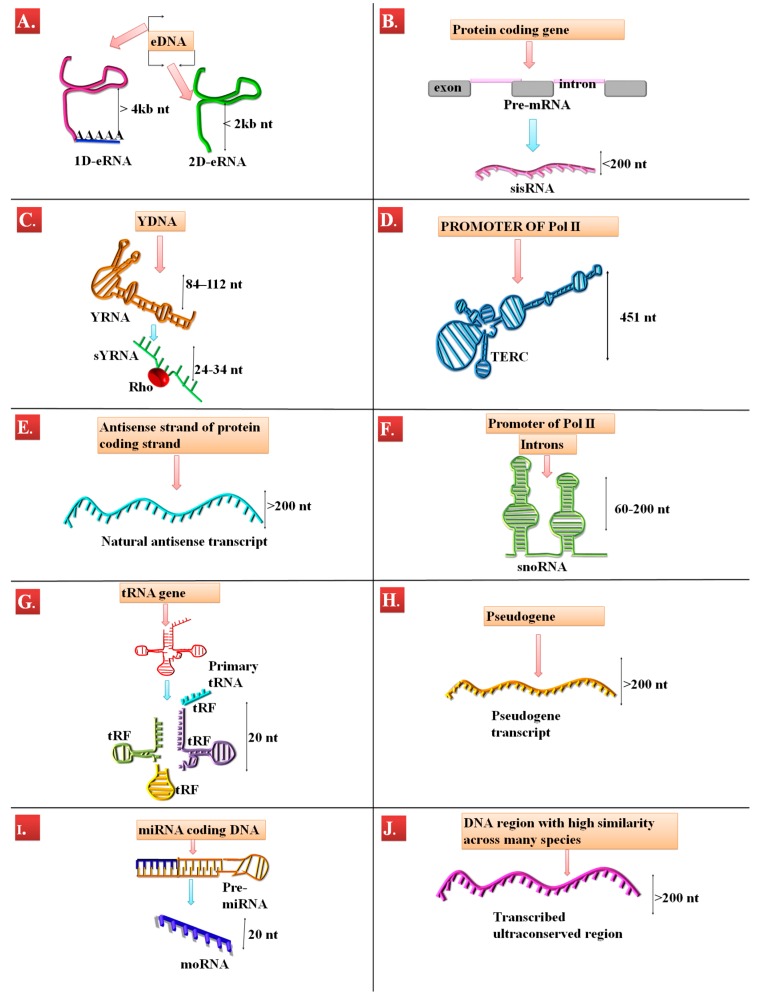
A schematic representation of the biogenesis and the main characteristics of some rare RNA species. The bidirectional arrow represents the length of the transcript, the red arrow stands for transcription and the blue arrow stands for RNA processing. (**A**) The enhancer RNA (eRNA) is transcribed from the enhancer DNA (eDNA) region. The transcription can be unidirectional; in this case, eRNA is polyadenylated, has a length of more than 4 kb and is named 1D-eRNA. If the eRNA is transcribed bidirectionally, it is not polyadenylated, has a length of less than 2 kb and is called 2D-eRNA. (**B**) The stable intronic RNA (sisRNA) is transcribed as primary messenger RNA (mRNA) transcript. After splicing, the intronic sequence remains stable thus forming the sisRNA. (**C**) The YRNA is transcribed from special regions of the DNA, called YDNA. The YRNA can remain in the nucleus or it can be cleaved into smaller fragments associated with Rho proteins. The smaller fragments are called small YRNA (sYRNA) and are sometimes exported into the cytoplasm. (**D**) From the promoter region of the RNA polymerase II (Pol II), the telomerase RNA component (TERC) is transcribed. TERC helps with telomerase activity. (**E**) From the antisense strand of protein-coding genes, the natural antisense transcript (NAT) is synthesized. NAT bears many similarities to the mRNA. (**F**) the small nucleolar RNA (snoRNA) can be originated from the intronic regions of protein-coding genes or from the promoter region of RNA Pol II. (**G**) From transporter genes, transfer RNA (tRNA) is formed; this transcript can be further fragmented and it forms the tRNA-derived fragments (tRFs). (**H**) The endogens are former protein-coding genes that have accumulated major mutations throughout evolution and become non-functional. The pseudogenes can be transcribed and the pseudogenes transcript bears many similarities to the mRNA. (**I**) The primary form of miRNA is processed by Argonaute (AGO) proteins. After processing, some part of the primary transcript remains in the nucleus, becomes stable and is very similar to miRNAs. These transcripts are named miRNA-offset RNA (moRNA). (**J**) Some regions of DNA, also considered non-functional, have high similarity across different species. These regions are called ultraconserved regions and can be transcribed. The transcribed ultraconserved region (T-UCR) is also similar in some aspects to mRNA.

**Figure 2:**
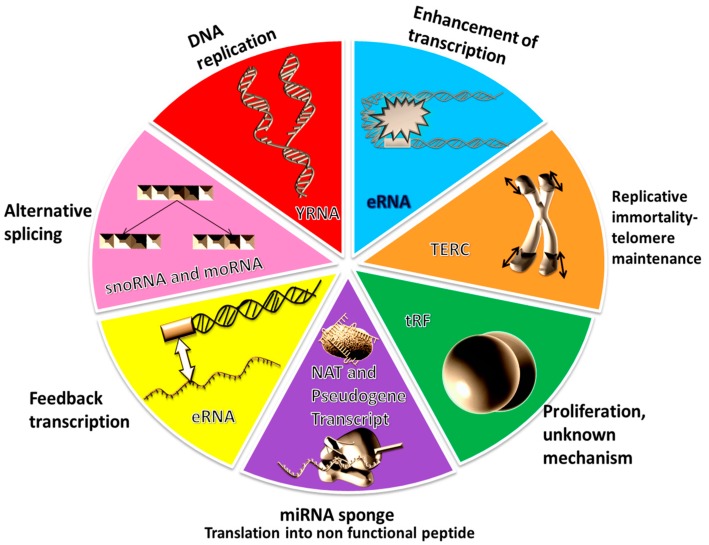
The lesser-known non-coding RNAs (ncRNAs) analyzed in this review have different mechanisms of action. NAT and pseudogene transcripts function as miRNA sponge, esiRNA and miRNA sources and can possess coding capacity. moRNA, T-UCR and sisRNA regulate gene expression through post-translational interaction with different coding or non-coding RNA species. In the alternative-splicing process, in addition to snoRNA, sisRNA might also play a role. YRNA is implicated in DNA replication and in deciding the fate of misfolded RNA. T-UCR and eRNA control gene expression before transcription: T-UCR by modulating the methylation pattern of CpG island and eRNA by chromatin structural changes and interaction with the transcription factors. TERC, through association with telomerase, maintains the length of telomeres, thus enabling replicative immortality. sYRNA were shown to be implicated in cell apoptosis, but the exact mechanism remains unknown. In addition, tRFs are involved in cell proliferation through an unknown mechanism.

miRNA-offset RNAs (moRNAs) are located in the nucleus [38], where they interact with messenger RNA (mRNA) [39,40]. Transcribed ultraconserved regions (T-UCR) have two mechanisms of action; changing the methylation of CpG island in the DNA [41,42] and miRNA inhibition through direct interaction [43,44,45]. The stable intronic sequence RNA (sisRNA) mechanism of action interacts post-transcriptionally with natural antisense transcript (NAT) in *Drosophila melanogaster* [46]. sisRNAs are also thought to regulate the expression of their own gene [47]. Some sisRNAs interact with the small nucleolar RNAs (snoRNAs)-specific protein FOX2, hence they are also involved in alternative splicing [48]. snoRNAs are involved primarily in ribosomal RNA (rRNA) nucleotide changes, but also in the splicing machinery [49]. YRNAs decide the fate of misfolded RNAs [50] or bind to the chromatin during the early stages of DNA replication [51]. Small YRNAs (sYRNAs), even if derived from the YRNAs, seem to have an unknown molecular mechanism of action. They sustain, however, the toll-like receptor-mediated cell apoptosis [52]. Enhancer RNAs (eRNAs) interact with transcription factors and the promoter region of their target gene in a specific spatial-temporal manner that results in the transcription stimulation [53]. Telomerase RNA component (TERC) interacts with telomerase and helps in telomere length maintenance [54]. In plants, it was proven that tRNA (transfer RNA)-derived fragments (tRFs) interact with the argonaute 1 (AGO1) and Dicer-like proteins and repress the transportable elements expression [55], but, in animal cells, their mechanism of action is still unknown [56]. NAT can act as source of endogenous siRNA, as post-transcriptional repressors for the mRNA of the antisense strand [57] and as modulator of heterochromatin [58]. The pseudogenes are transcribed in approximately 20% of cases. Some pseudogene transcripts were even found to be translated into peptides [52,59,60]. Whether these peptides have a functional role inside the cell remains to be studied [60,61]. On the other hand, pseudogene transcripts can also have regulatory roles inside the cell. They can be sources of endogenous siRNA and miRNA [62], can sponge miRNAs [63] or regulate gene expression through epigenetic modifications [62]. Moreover, recently, based on bioinformatics tools, it was found that small RNAs transcripts interact with long non-coding RNAs (lncRNAs) [64,65]. The main characteristics of these transcripts are summarized in Table 1. Their biogenesis is illustrated in Figure 1 and their mechanism of action in Figure 2.

It is worth noting that some ncRNAs are located in the intronic region of the mRNA and that they are involved in splicing. This correlates with the observation that some synonymous mutations, located in the intronic region of the DNA, can cause translation repression or intronic maintenance in the transcript. This “intronic retention” was observed in several tumor suppressors transcripts in human cancer [66].

## 3. Enhancer RNAs 

eRNAs were initially described in neuronal cells by Kim et al. in 2010, by using genome-wide sequencing approaches [82]. eRNAs are a type of ncRNAs of 50 bp–2 kb length that reside in the nucleus and are transcribed from the enhancer DNA. The enhancer DNA represents various areas of the DNA with the main characteristic of controlling gene expression. The enhancers interact especially with Lineage Determining Transcription Factors (LDTFs). LDTFs determine a cell type and act by modifying the chromatin structure through histone methylation. A chromatin loop is formed that allows the interaction between the enhancer, the transcription factor and the promoter region of the targeted gene. eRNAs are produced to facilitate loop formation. eRNAs arise as unidirectional (1D-eRNAs) or bidirectional transcripts (2D-eRNAs) [83]. Generally, 1D-eRNAs are long (over 4 kb) and polyadenylated, while 2D-eRNAs are relatively short (<2 kb) and are not polyadenylated [84,85,86,87]. eRNAs play crucial roles in pathogenesis [82]. The basal cell carcinoma is closely linked to the *ACTRT1* gene transcript ARP-T1 [88]. The Cancer Genome Atlas (TCGA) data analysis present molecular data, including those related to eRNAs, for different types of cancer. It was demonstrate that the metastatic niche preparation is reflected by the differentially methylated enhancer regions, followed by stimulated transcription of oncogenes and oncomiRs [89]. Enhancer RNAs can be *cis*-regulators and act on the same chromatin fiber or *trans*-regulators and induce the transcription of a gene found on another chromosome [87]. An interesting finding was the fact that p53 protein was bound mostly in the enhancer regions of the DNA. As follows, p53 modulates the expression of eRNA, in human and mouse primary fibroblast [90]. The p53 binding regions and the dysregulation of eRNAs in head and neck neoplasia may constitute a fertile ground for further studies [31,91]. 

## 4. Stable Intronic Sequence RNAs 

SisRNAs are a class of ncRNAs originated from the spliced introns that are not degraded by the nucleases [92]. Previous studies have postulated their existence, but a study from 2012 was the one that has offered the best description yet [92]. sisRNAs are transcribed from the protein-coding genes as primary mRNA transcripts. After splicing, part of the remaining intronic RNA remains stable in the nucleus for days [92], which is why these RNA species were given the name of stable intronic sequence RNA. After the lariat is formed during splicing, the intronic sequence can remain in the circular form as circular sisRNAs (also known under the name of circular intronic RNA) or the 2′–5′ phosphodiester bond can be cleaved by the lariat debranching enzyme, and the linear sisRNA is generated [47]. In H9 and HeLa cells, it was discovered that the sisRNA ci-ankrd52 can bind to the Pol II and stimulate the transcription of their own gene of origin [70]. A linear sisRNA directs the activation induced cytidinedeaminase (AID) enzyme in the DNA of B cells that helps the immunoglobulin class switch [71]. A linear sisRNA regulates the alternative splicing process in pluripotent cells by interacting with the FOX2 protein [48]. Epstein-Barr virus (EBV) presence was correlated among other cancers, to oral squamous cell carcinoma [93]. A RNA-Seq analysis in EBV transcriptome reveal the presence of a sisRNA, ebv-sisRNA-1 [94].

## 5. YRNAs

YRNAs were sequenced and characterized in 1980 [95]. YRNAs are versatile transcripts involved in small RNA quality control, replication mechanism and are related with cellular processes that sustains cell proliferation. They are also synthesized in response to cellular stress [96]. In addition, YRNAs are transcribed from a special part of the DNA, called YDNA. They have a stem loop structure and a length of approximately 84–112 nt. As such, there are four types of yRNAs in humans, hY1, hY3, hY4 and hY5, which have one thing in common, i.e. they are bound to the Ro60 and La proteins [97,98]. These ncRNAs are encoded by the *Y* genes located in close proximity of one another and the polymerase III (Pol III) is responsible for their transcription [72] in the form of a stem-loop secondary structure [99].

YRNAs are recognized as a component of soluble ribonucleoproteins (RNPS). These are related with many pathological conditions [100]. The Ro60 protein bound to the YRNAs controls the fate of the misfolded RNAs. The misfolded RNAs are refolded or degraded [50]. YRNAs are also a part of the initial phases of DNA replication by interacting with the chromatin and some of the replication proteins, such as Cdc6, Cdt1, DUE-B and ORC [72]. 

These small RNAs were, for a long period of time, categorized as miRNAs, but now they are considered as a subtype of yRNAs [101,102]. sYRNAs are particularly present in apoptotic cells. sYNAs were initially discovered in tumor tissue. These RNAs are not processed by Dicer and do not interact with the Ago proteins [98]. The sYNAs have a size range of 25–29 nt [103]. Their biological functions are still largely unknown. In primary cell culture of macrophage from C57BL/6 J mice and THP.1 monocytes, RNY1 or RNY3 was related with the apoptosis activation through the cleavage of caspase-3 and NFkB signaling pathway [74]. Several sYRNs were found to be down-regulated in the sera of head and neck cancer patients, compared to healthy donors, 30.2% vs. 44.2%. Using next generation sequencing (NGSseq) approaches, a specific yRNA was identified along with miRNA and tRNA signature related to head and neck squamous cell carcinoma (HNSCC), revealing a complex regulation of oncogenic processed. The most relevant altered yRNAs transcripts in head and neck cancer are summarized in Table 2 [104]. In addition, sYRN an apoptotic regulator of apoptosis was proposed as diagnostic marker [105]. s-YRNs can be detected in tandem with other short transcripts length transcripts leading to the identification of novel signature with clinical application [105].

## 6. The Human Telomerase RNA Component or Human Telomerase RNA 

The telomerase enzyme comprises two crucial constituents that operate together. The first one represents a reverse transcriptase (TERT) that has a catalytic unit associated and the other one is the TERC. TERC is a RNA template from which the missing part of the DNA is synthetized [106]. TERC was first discovered in eukaryotic cells. The human TERC (hTR) is overexpressed in the germline and in the cancerous cells [107].

hTR contains two hairpin structures separated by an H box and a single stranded ACA box. hTR is encoded by a sequence located in the promoter region of Pol II gene. Following the production of a primary transcript that has 1451 nt, the cap-binding complex (CBC) links with the ARS2 protein and directs the hTR towards processing or degradation [54]. The nuclear exosome targeting (NEXT) complex is formed of several proteins which recognize alterations in the human telomerase primary form, such as 3′ extension or oligoadenylation. The NEXT complex mediates the hTR degradation by the nuclear RNA exosome complex [54]. The nuclear RNA exosome complex is a multi-protein complex that is involved in the quality control of several transcripts [108]. The final hTR transcript has 451 nts [54,75]. It was proven that it is possible for the hTR to be exported into the cytoplasm. The exported hTR was either degraded by the XRN1 and DCP2 enzymes, or multiple hTR formed complexes called cytoplasmic TERC (cyTERC) [109]. 

The hTR is upregulated in oral cancer tissue from patients and was associated with the malignancy installment. With the help of reverse transcription polymerase chain reaction (RT-PCR), an over 6.9-fold increase in the expression of hTR in oral squamous cell carcinoma was proven, when compared to the normal tissue. It seems that the expression of this RNA increases with the malignant transformation of cells, since it had low levels in normal epithelium and mild dysplasia, higher in moderate and severe dysplasia and the highest in the invasive oral carcinoma [110]. The Fragile *X*-related protein 1 (FXR1) is upregulated in HPV-negative tongue squamous cell carcinoma cell lines UMSCC74A and UMSCC74B, in which it was found to play an essential role in cell cycle progression and senescence evasion. One way in which FXR1 can cause this pathological shift is through the interaction and stabilization of hTR. The induced overexpression of hTR in these cell lines caused an amplification of immortality [111,112].

## 7. Natural Antisense Transcripts 

NATs were discovered a few decades ago [113]. Some of them function as protein-coding RNAs and others as ncRNAs. They are transcribed from the antisense strand of a protein-coding gene located at the same DNA locus (cis-NAT) or from a distant site of the DNA (trans-NAT) [114]. NAT has a full complementarity with the protein-coding strand. It can stimulate or repress the transcription of a gene by modulating the heterochromatin interaction [58] or by functioning as a source of endogenous small interfering RNAs (esiRNAs) [57].

WRAP53 (WD40-encoding RNA antisense to p53) is transcribed from the antisense strand of p53 gene. It can interact with the p53 mRNA and repress its translation. Through real-time quantitative reverse transcription PCR (qRT-PCR) and Western blotting, the WRAP53 NAT was detected to have an elevated level in the esophageal carcinoma tissue samples and in the cell lines EC109, EC9706, KYSE150, and KYSE180. Its expression increases with disease progression and it was associated with the lymph node metastasis [76]. These natural antisense transcripts to p53 play a significant role in the development and progression of oral squamous cell carcinoma (OSCC), representing an important therapeutic target or biomarker [76]. It was demonstrated that ATM it is an upstream regulator of these transcript, being implicated not only in DNA damage response, but in the regulation of other functions as well [115].

## 8. The Small Nucleolar RNAs 

snoRNAs are a type of linear transcripts with protein-guiding function. Depending on their sequence characteristics, the snoRNAs are divided into C/D snoRNA, H/ACA snoRNA, and U snoRNA [116,117]. The C/D snoRNA and the H/ACA snoRNA are transcribed from protein-coding genes, as primary mRNAs. After splicing, the intronic lariat is cleaved into a linear form by the Dbr1p protein. Then the 3’ and the 5’ end are shortened by the local endonucleases, until the mature C/D or H/ACA snoRNA is generated. The U3, U8 and U13 snoRNAs are transcribed from the promoter regions of RNA Pol II [116].

The mature forms of snoRNAs have the sequence length of 60– 300 nt [118]. Based on their structure, the snoRNAs are classified into C/D box snoRNAs and H/ACA box snoRNAs. The C/D box RNAs have a double C box (RUGAUGA, R = A/G) and a double D box (CUGA). They are arranged in the C-D-C-D order and bind specific proteins, such as Snu13p, Nop56p, Nop58p and fibrillarin [119,120]. A small subclass of C/D snoRNAs has only the C or the D box and it has been reported to be subjected to mutation accumulation across species [120]. The H/ACA snoRNAs contain the H sequence (ANANNA, N = A/T/C/G) and the ACA sequence and two stem regions. These H/ACA boxes mediate the association with Gar1p, Cbf5p, Nhp2p, and Nop10p proteins [118,119]. Generally, snoRNAs are involved especially in rRNA processing. The C/D snoRNAs cause 2′-*O*-methylation of rRNA. The H/ACA snoRNAs convert rRNA uridine into pseudouridine [49,119]. These ncRNAs, such as HBII-52, are also components of the splicing machinery [49]. 

Apart from these functions there are several snoRNAs whose functions remain unknown. They are called orphan snoRNAs. Orphan SNORD32A (U32A), SNORD33 (U33) and SNORD35A (U35A) accumulate in the cytosol under lipotoxic and oxidative stress, hence it was theorized that snoRNAs can be exported in the cytoplasm and fulfill other roles [78]. 

By analyzing the TCGA RNA sequencing (RNASeq) data, it was discovered that snoRNA SNORD35B was upregulated in some head and neck cancer and its expression was positively associated with a lower survival rate among the patients. The overexpression of this snoRNAs was confirmed by qRT-PCR in the cell carcinoma cell lines UMSCC-10B, UMSCC-22B, HN-1, HN-12, and HN-30 [121]. In another subtype of rare oral cancer named ameloblastoma, several snoRNAs were found to be dysregulated. LINC340, SNORD116-25, SNORA11, SNORA21, SNORA47 and SNORA65 were proposed as biomarkers [122].

**Figure 3:**
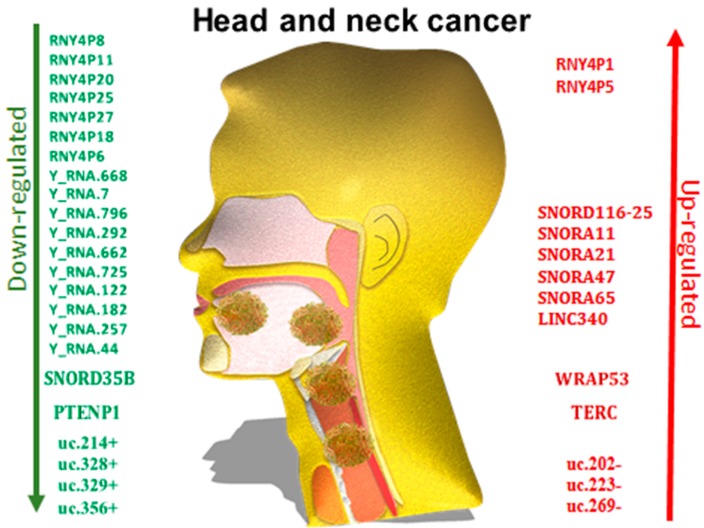
A short list of the most studied down-regulated and up-regulated rare ncRNAs in head and neck cancer discovered to this date.

The microarray comparison made between OSCC tissue and keratinized gums, proved that 14 snoRNAs were down-regulated (displayed in Figure 3) and one snoRNA was up-regulated in OSCC. An accentuated down-regulation of mgh18S-121 and ACA17 can be the cause of a worse prognostic [123].

## 9. tRNA Derived Small RNAs 

tRFs are transcripts with regulatory role, originated from tRNA. They are 20 nt in length and are divided into nine categories, depending on their biogenesis and mechanism of action. There are specific RNase which cleaves the primary tRNA transcript. At the tRNA 5′ end, cleavage is catalyzed by the ubiquitous endoribonuclease RNase P, and tRNA 3′ end cleavage is specific for RNase P [124,125].

The cleaved fragments, that have poly-U repeats at the 3’ end, are categorized as tRF-1. The Dicer enzyme may further process the mature tRNA1 by a cut in the T loop and generate the tRF-3. The tRf-3 has a CCA sequence at its 3’ end as a remnant of the original tRNA acceptor sequence. The mature tRNA can also be cleaved by the angiogenin enzyme and give rise to the tRF-5 [56,126]. 

The tRFs biological function is still unidentified, but until now, studies indicate that it may have a profound role in cancer. The tRF-1001 is responsible for increased cell proliferation [56]. They are also dysregulated in breast cancer and B cell lymphoma [127]. To the best of our knowledge, no study was done regarding the evaluation of these RNAs in head and neck cancer. 

## 10. Pseudogene Transcripts

Pseudogenes are paralogous to coding genes and are the result of duplication associated with mutation. Because they are lacking the promoter and regulatory regions of the coding genes, for a long time, pseudogenes were viewed only as vestigial sequences without any function [128]. However, during the latest studies, it was proven that the pseudogenes are transcribed and that their codified transcripts are a new type of RNAs which can either regulate other RNA or are translated into proteins. Pseudogene RNA can cause epigenetic modifications of the genome by interacting with the chromatin folding around histones. They can be processed into miRNAs or can sponge other miRNAs. 

Pseudogene transcripts are a source of esiRNAs that targets their paralogous genes. There are pseudogenes regulated by the same promoter as the protein-coding gene. These can be transcribed and translated into a chimerical protein [62,79]. The pseudogene transcripts ability to be translated was proved through proteomic analysis from human tissue [52], however whether these proteins fulfill any function remains to be determined [61]. Because of their length, pseudogene RNA are sometimes regarded as lncRNAs [79]. 

In head and neck cancer, it was proven that lncPTENP1, which is in fact a pseudotranscript of the *PTEN* gene, is under-expressed in the HN4, HN6, HN13, HN30 and Cal27 cell lines, being associated with lower survival rate in HNSCC patients. Through lentiviral transfection and restoration of the expression level of PTENP1, it was proven that, in vitro, the cells had decreased proliferation, invasion and migration capabilities, while, in vivo, they failed to form tumors [62]. The PTENP1 pseudogene transcript can bind the same micro-RNAs as the original gene *PTEN*, more precisely the miR-17, 21, 214, 9, and 26 families and act as a decoy mechanism that allows the translation of the *PTEN* gene [129]. The pseudogenes have *cis-* regulating sequences that compete with the *trans*-regulators of the protein-coding mRNAs [130].

Some rare ncRNAs are transcribed from pseudogenes. For instance, apart from the 5 YDNA genes located in the proximity of one another, it was reported that there are some YDNA pseudogenes that are spread throughout the whole genome. These pseudogenes existence was probably due to the retrotransposition of *trans*-mutated human YRNAs by the long-interspersed element-1 (L1). These hY-pseudogenes (hYRNAs) are also transcribed into a novel type of human yRNAs that may add a higher degree of complexity to the five hYRNAs [131].

## 11. miRNA-Offset RNAs 

moRNAs were discovered in the chordate *Ciona intestinalis* through small RNASeq in 2009 [132]. They are enriched in the nucleus, being related with the regulation of nuclear processes, particularly transcription initiation and splicing activity [133]. moRNAs are similar in length and structure with miRNA. They have a length close to 20 nt. The biogenesis mechanisms are related to Drosha/DGCR8 complex that processes the multiple stem-loops of pri-miRNA transcript into the stem-loop form of pre-miRNA. The sequences that were thought to be discarded are kept in the nucleolus, giving rise to moRNAs [80].

With the small RNAseq technique, which compared the human embryonic stem cells with the normal fibroblast cell line, it was found that 326 moRNAs were in stem cells compared with 65 found in normal fibroblasts. The most abundant human moRNAs include moR-367-3p, moR-103a-2-3p, moR-92a-1-3p, moR-16-2-3p, moR-21-3p, moR-221-3p, moR-103a-1-3p, moR-20b-3p, moR-421-5p, and moR-21-5p [80]. Although their exact role in oral cancer remains unknown, they have potential to become well-studied ncRNAs because of their similarity with miRNAs. 

## 12. Ultraconserved Regions of the DNA 

UCR are sequences of the genome located in the exonic, intronic or even intergenic regions. In 2007, through microarray analysis, it was discovered that these UCRs are transcribed, giving rise to a new type of long non-coding RNAs, called T-UCRs [43]. They are highly similar across species and can bear up to 100% similarity in the same species. Surprisingly, it was discovered that the T-UCRs are dysregulated in multiple human cancers, such as bladder cancer, colorectal cancer, hepatocellular carcinoma, prostate cancer and chronic lymphocytic leukemia (reviewed in [81]). 

In an esophageal cancer subtype, Barrett’s adenocarcinoma, T-UCRs were evaluated. The down-regulated T-UCRs, i.e., uc.214+, uc.328+, uc.329+ and uc.356+, and up-regulated T-UCRs, i.e., uc.202−, uc.223−, and uc.269−, were found to be associated with the carcinogenesis of this malignancy through tissue microarray analysis [134].

All the above mentioned new information about the lesser-studied transcripts has impacted cancer research. The pseudogene transcripts are dysregulated in the head and neck malignancies (Figure 3 and Table 2), suggesting that they can become valuable diagnostic, prognostic or even therapeutic markers [62]. 

## 13. Exosomes as Important Sources of Rare ncRNAs

Exosomes are small vesicles of 40–100 nanometers that can carry information from one cell to another [136]. These small vehicles are important due to their cargo content [137,138]. They carry miRNAs [139], siRNAs [140], mRNA and DNA fragments [141]. 

It was discovered that the exosomes can also carry yRNAs associated with small fragments of mRNA thus proving the fact that the initial stages of exosomes formation can include the whole machinery of RNA degradation [142]. The extracellular vesicles were proven to be loaded with a greater amount of RNY5, in different cancer cell lines [143]. In fact, the YRNA concentration in exosomes was detected to be in a greater quantity than in the intracellular environment. The RNAY5 is the second most abundant RNA species in the human epithelial cell line and breast cancer cell line [144]. In the exosomes, RNY1 and RNY4 were also found [142]. The small YRNA serum levels in healthy donors was comparable to that of miRNAs [73].

By using LRRC24, MDM2, and CDKN1A primers in a RT-PCR analysis, after RNA isolation from colorectal cancer cells-derived exosomes, it was established for the first time that the exosomes can also carry NATs [145]. Another important class of ncRNAs found in the exosomes were snoRNAs, compared even with their profile from the cell of origin [142]. 

**Figure 4:**
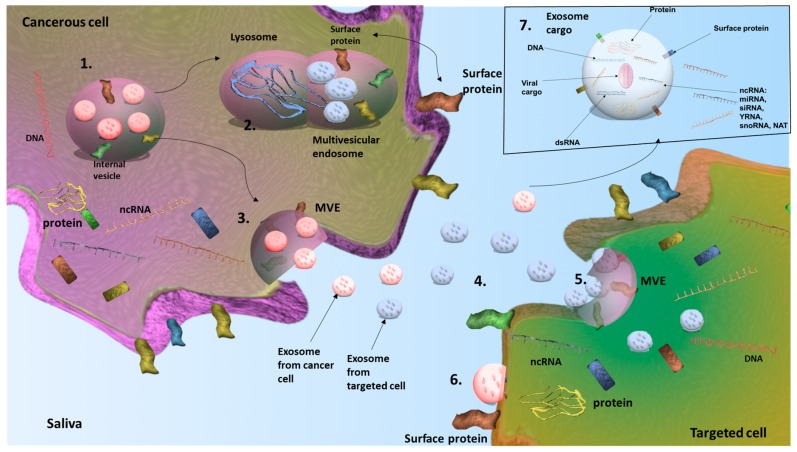
Salivary exosome-mediated intracellular communication in oral cancer. (**1**) In the tumor cell, by the budding of membrane, the multivesicular endosome (MVE) is formed, containing small vesicles of 40–100 nm called exosomes. The MVE can have an endogenous or an exogenous origin. (**2**) The MVE can fuse with a lysosome and its content is destroyed. (**3**) The MVE can also fuse with the plasma membrane and release the exosomes in the saliva. (**4**) The exosomes circulate through the body fluids such as the saliva or the blood and reach a targeted cell. (**5**) In the targeted cell, the exosomes are recognized by the surface proteins and are internalized through endocytosis. (**6**) The exosome can also bind directly to the plasma membrane and release its content into the cytoplasm. (**7**) An exosome contains surface proteins necessary for the engulfment by the targeted cell. In its interior, the exosome contains ncRNAs, DNA fragment and other proteins. This cargo can influence the behavior of their targeted cell. The exosome can contain also viruses; hence, it can mediate the spreading of human papilloma virus or Epstein-Barr virus.

Exosomes are also secreted in the saliva and the molecular profiling of exosomes was proposed as a better option for evaluation of oral health status (Figure 4), diagnosing oral cancer and assessing the progression of this malignancy [138,146]

A focus should on the cargo content related to viral exposure such as HPV or EBV, which is a predisposition factor for some cancer types. These small salivary vesicles can be used as an alternative model in biomarkers discovery.

Although currently exosomes from saliva are evaluated mostly for their miRNA and siRNA cargo, the future analysis of salivary exosomes can also examine their cargo of the lesser-studied ncRNAs. 

## 14. Conclusion and Perspective

Based on past experience with siRNA and miRNA, the current knowledge regarding the ncRNA world could see dramatic changes in the following years, by increasing the importance of these rare transcripts in the clinical application. However, for sYRNA, snoRNA, NAT and T-UCR involvement in head and neck cancer, data are scarce. As for the others ncRNAs, such as eRNAs, sisRNAs, tRFs and pseudogene transcripts, there are indications of their dysregulation in other cancer types. 

Notwithstanding the fact that there is a great diversity of studies about ncRNAs, we have to bear in mind that it is still in its infancy. Consequently, there is yet much to be learned about the sources of ncRNAs; their various maturation steps; mechanism of action; and interactions with the DNA, proteins, other coding or ncRNAs, all of which could offer the scientific community a better view of head and neck cancers and new ideas for oncological personalized therapy. These are sustained by the latest progress related to the approaches used for the evaluation.

## Figures and Tables

**Table 1:** The main features of less known non-coding RNAs (ncRNAs).

ncRNA Type	Length (nt)	DNA Region	Mechanism of Action	Reference
eRNA	800–2000	enhancer DNA	histone methylation	[67,68,69]
sisRNA	intronic length	protein-coding genes	alternative splicing, transcription stimulation, antibody isotype class switch	[70,71]
YRNA	84–112	yDNA	misfolded RNA degradation, DNA replication	[50,72,73]
sYRNA	~24 to ~34	yDNA	interaction with TLR	[74]
TERC/hTR	451	Pol II promotor region	telomere maintenance	[54,75]
NAT	depending of the coding gene length	antisense strand of protein-coding genes	mRNA inhibition	[76]
snoRNA	60-200 C/D snoRNAs, 120–250 for H/ACA snoRNAs	introns, promoter region of Pol II	rRNA processing, splicing	[77]
tRF	20	tRNA coding genes	Unknown	[78]
Pseudogenestranscrips	gene lenght	Pseudogenes	translation repression, miRNA sponge, multiple miRNAs, siRNA origin	[62,79]
moRNAs	20	miRNA-coding genes	Unknown	[80]
T-UCR	>200	Ultra-conserved regions of the DNA	miRNA sponging	[81]

eRNA: enhancer RNAs; nt: nucleotides; sisRNA: stable intronic sequence RNAs; sYRNA: small YRNA; TLR: Toll-like receptors; TERC: telomerase RNA component; hTR: human telomerase RNA; Pol II: polymerase II; NAT: natural antisense transcript; mRNA: messenger RNA; snoRNA: small nucleolar RNAs; H/ACA: specific H and ACA template boxes; rRNA: ribosomal RNA; tRF: tRNA-derived fragments; tRNA: transfer RNA; miRNA: micro RNA; siRNA: small interfering RNA; moRNAs: miRNA-offset RNAs; T-UCR: transcribed ultraconserved regions.

**Table 2:** Rare types of ncRNAs retrieved to be dysregulated in head and neck cancer.

Type of ncRNA	Name/Code	Expression	Determination method	Clinical/In Vitro/In Vivo	Observation	Reference
syRNAs	RNY1, RNY4P17	Up	Deep sequencing with IlluminaHiSeq 2000	Clinical blood samples	Altered expression level in head and neck cancer	[104]
	RNY4P1, RNY4P5 RNY4P8, RNY4P11 Y_RNA.725, Y_RNA.122 RNY4P20, RNY4P25 RNY4P27, Y_RNA.182 Y_RNA.257, RNY4P18 Y_RNA.44, RNY4P6 Y_RNA.668, Y_RNA.7 Y_RNA.796, Y_RNA.292 Y_RNA.662	Down				
TERC/hTR		Up	qRT-PCR	in vitro UMSCC74A, UMSCC74B/TCGA	cell senescence inhibited	[111]
		Up	Gene copy number—FISH	OSCC—tissue samples vs. normal tissue	installment of oral lesions	[135]
		Up	RT-PCR	oral cancer tissue vs. normal tissue	associated with higher grade dysplasia or carcinoma	[110]
snoRNAs	SNORD35B	Down	RNA-Seq for lncRNA, (TCGA)/RT-PCR	in vitro normal cell lines OKF4 and OKF6 vs. oral cancer cell lines UMSCC-10B, UMSCC-22B, HN-1, HN-12, and HN-30	associated with poor prognostic	[121]
					mediate the pattern of 28S rRNA	
	4qI-4,14qII-22,ACA17,U84, mgh18S-121, U18A, U8, 14qII-12, U28 ENSG00000263442, ENSG00000264591, ENSG00000265325, ENSG00000265607, ENSG00000266646, ENSG00000266755,	Down	Microarray	OSCC tissue vs. keratinized gums preserved at −80 °C	Potential biomarkers, prognostic value	[123]
	LINC340, SNORD116-25, SNORA11, SNORA21, SNORA47 and SNORA65	Up	microarray, RT-PCR	Formalin fixed and paraffin embedded (FFPE) samples of ameloblastoma clinical	better diagnostic tool through biomarkers and potential therapeutic targets	[122]
NATs	WRAP53	Up	qRT-PCR, Western-blot	ESCC cell lines EC109, EC9706, KYSE150, and KYSE180 and tissue from 134 oesophageal cancer patients	higher expression of this transcript is progression of oesophageal cancer	[76]
Pseudogenes transcript	PTENP1	Down	HN4, HN6, HN13Cal27, HN30 cell lines		proliferation, invasion and migration capabilitiesand decreased survival rate	[37]
T-UCRs	uc.214+, uc.328+, uc.329+, uc.356+,	Down	Microarray, RT-PCR, immunohistochemistry	oesophageal cancer	cancerous transformation	[134]
	uc.202-, uc.223-uc.269-	Up				

qRT-PCR: real-time quantitative reverse transcription PCR; FISH: fluorescent hybridization in situ; OSCC: oral squamous cell carcinoma; RT-PCR: reverse transcription polymerase chain reaction; lncRNA: long non-coding RNA.
